# A Retrospective Study Characterizing the Radiographic Progression of Hallux Valgus

**DOI:** 10.7759/cureus.24607

**Published:** 2022-04-29

**Authors:** Vasil V Kukushliev, Alex T Burton, Glenn G Shi, Brian C Law, Jonathan C Kraus

**Affiliations:** 1 Department of Orthopaedic Surgery, Medical College of Wisconsin, Milwaukee, USA; 2 Department of Orthopaedic Surgery, Mayo Clinic, Jacksonville, USA

**Keywords:** hardy clapham, intermetatarsal, metatarsophalangeal joint, ankle and foot, hallux valgus

## Abstract

Background: Hallux valgus deformity is the lateral deviation of the metatarsophalangeal (MTP) joint and is the most common pathology of the great toe, affecting 2%-4% of the population. It is commonly believed that the condition progresses over time both in the magnitude of deformity and development of arthritic changes. To our knowledge, there are rare studies describing the rate of deformity progression and the development of arthritic changes. Our aim is to quantify the progression of hallux valgus and associated arthritic changes in an adult population using radiographs.

Methods: Patients who are 18 years of age and older (mean age: 61.7 years and range: 18.6-94.2) who presented to our institutions between January 1, 2004, and December 1, 2019, were included. Patients were included in the study if they had hallux valgus on weight-bearing radiographs and two such radiographs at least six months apart. Hallux valgus angle (HVA), intermetatarsal angle (IMA), metatarsophalangeal (MTP) arthritis, tarsometatarsal (TMT) osteoarthritis, Hardy-Clapham sesamoid position, and round sign were collected per patient in the initial and final radiographs. Included cases were first studied together in a whole group. Then, cases were separated into three groups based on the time between initial and final radiographs.

Results: A total of 52 radiographic records for 43 patients were included. HVA and IMA progress with time; however, progression does not follow a direct relationship with the time elapsed between initial and final radiographs (p = 0.92 and p = 0.35, respectively). The progression of TMT osteoarthritis, sesamoid position, and round sign do not show a dependence on the time elapsed (p = 0.20, p = 0.11, and p = 0.42, respectively). An increase of one sesamoid unit position over baseline is associated with a 0.85-degree increase in IMA. A one-unit progression of sesamoid position at baseline raises the odds of MTP osteoarthritis progression at the follow-up visit by 2.14 (OR = 2.14, p = 0.0007, CI = [1.35, 3.83]). A HVA increase of one degree increases the odds of TMT osteoarthritis progression at follow-up by a factor of 1.17 (OR = 1.17, p = 0.0005, CI = [1.07, 1.34]). Patients with MTP arthritis at the initial visit have 3.77 times higher odds of round sign progression on their follow-up visit (OR = 3.77, p = 0.027, CI = [1.16, 13.13]).

Discussion: Hallux valgus progression can be quantified. Upon their first visit, a patient’s hallux valgus parameters can be utilized to demonstrate expected progression. Progression of the deformity and arthritic changes is slow. Nonetheless, the results should be considered by surgeons and patients when developing a treatment plan with patients.

## Introduction

Hallux valgus is a deformity characterized by the abduction of the hallux with adduction of the first metatarsal [1-4]. This condition is the most common pathology of the great toe and affects 2%-4% of the population [5]. In some cases, the altered first metatarsophalangeal (MTP) joint alignment leads to impaired gait [3]. The causes of hallux valgus are plentiful and not entirely agreed upon [6]. Likely contributors can be split into intrinsic and extrinsic factors. Intrinsic factors are genetics, sex (female > male), ligamentous laxity, other foot deformities (e.g., pes planus, hindfoot pronation, and metatarsus primus varus), age, and neuromuscular disorders (e.g., cerebral palsy and stroke). Extrinsic factors include significant external forces acting on the great toe for prolonged periods, for example, footwear such as high heels or narrow shoes [7].

A constellation of changes occurs with the progression of the condition, such as the deviation of the metatarsal head medially, pronation of the metatarsal and hallux, subluxation of the metatarsal head away from the sesamoid, instability in the midfoot, and degenerative changes in the MTP, sesamoid, and TMT joints [6].

Patients with hallux valgus typically present with pain over the medial eminence, MTP joint pain, intolerance of shoe wear, bursal or skin irritation, and ulceration or infection [5,8]. Physical exam findings include medial deviation of the first metatarsal, lateral deviation of the hallux, pronation of the hallux, and splay foot [3,5,9]. Physical exam findings possibly related to hallux valgus include arch pathology, first ray hypermobility, and a tight Achilles [5,8-10]. Articular shape, metatarsal length, prominence of medial eminence, joint congruity, distal metatarsal articular angle, and metatarsus adductus are disputed findings in hallux valgus presentation [5,9,11]. Due to the variability in presentation and certain disputed findings, abiding by the established radiographic classification of hallux valgus is a must.

Hallux valgus severity is quantified using radiographic measurements: specifically, the hallux valgus angle (HVA), the intermetatarsal angle (IMA), and, for certain patients, the distal metatarsal articular angle (DMAA) measurements [12]. Hallux valgus is defined by an increased HVA, increased IMA, and a change in the sesamoid position [5]. Metatarsophalangeal (MTP) arthritis, tarsometatarsal (TMT) osteoarthritis, Hardy-Clapham sesamoid position, and round sign have also been used as markers for hallux valgus progression [13-16]. Observation studies of these measurements have suggested that hallux valgus pathology progresses over time, except in cases of severe hallux valgus [12]. Severity is also subjected to patient-specific levels of disability that cannot be entirely represented via objective methods.

Hallux valgus progression is difficult to garner due to the many factors mentioned above that play a role. Nonetheless, evidence exists that there is a progression in the disease from studies showing the change between two separate time points [12,17]. To the best of our knowledge, there are only rare studies describing the rate of deformity progression and the development of arthritic change. Our aim is to utilize radiographs to quantify the impact that the variables mentioned above have on disease progression and to determine the rate of progression. Our work was presented at the 2022 Orthopaedic Research Society Annual Meeting.

## Materials and methods

Following IRB approval, a retrospective study ensued identifying patients who are 18 years of age and older who presented to our institutions between January 1, 2004, and December 1, 2019. A text search of the radiographic interpretation was done to include any terms that are mapped to symptomatic hallux valgus. These patients had presented to our institution with concerns about hallux valgus. Charts were included only if a patient had two or more films greater than six months apart. A total of 331 charts were initially identified that met these parameters. Patients were excluded if the films did not include weight-bearing views, if there was evidence of prior foot surgery, or if patients had rheumatoid arthritis. Our study considered the amount of time between initial and final radiographs, and it was also noted if a patient eventually underwent surgical intervention after their final radiograph.

Measurements were taken using the Picture Archiving and Communication System (PACS). Radiographic evaluation was performed by two authors, J.K. and A.B., who analyzed the initial and final radiographs of all patients included in the study. A total of 52 radiographic records for 43 patients were included, with nine patients providing bilateral images and 34 providing unilateral images. HVA, intermetatarsal angle (IMA), metatarsophalangeal (MTP) osteoarthritis, tarsometatarsal (TMT) osteoarthritis, Hardy-Clapham sesamoid position, and round sign were collected from the initial and final radiographs for each participating case.

**Figure 1:**
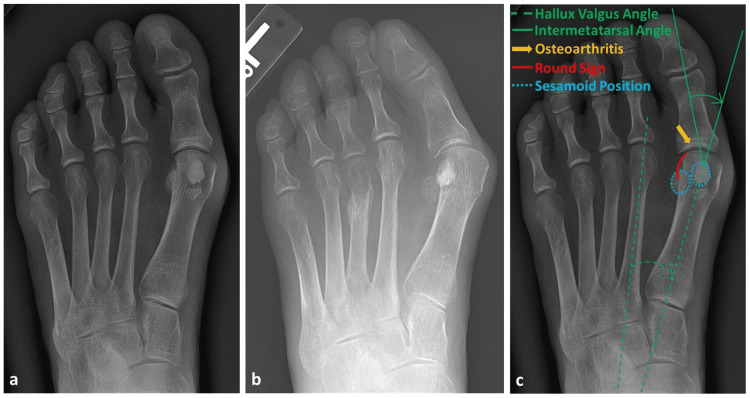
Radiographs of a 54-year-old female patient (a) An initial encounter radiograph and (b) an 85-month follow-up radiograph. Increases in HVA, IMA, osteoarthritis, Hardy-Clapham sesamoid position, and round sign are observed. Labeled measures (c) are provided in the initial encounter radiograph.

HVA was captured by measuring the angle between the midlines of the first and second metacarpals. IMA was captured by measuring the angle between the midlines of the first metacarpal and the first proximal phalanx. Osteoarthritis was qualitatively evaluated on whether it was present to begin with and whether it had progressed or appeared on the final radiograph. The Kellgren and Lawrence scale (0-4) was utilized to evaluate osteoarthritis [18]. For sesamoid position measurements, Hardy and Clapham’s tibial sesamoid 7 position system was utilized [2]. Furthermore, the round sign was qualitatively evaluated through the roundness of the lateral perimeter of the first metatarsal head [16,19]. Sample radiographs and observed measurements are provided in Figure 1.

All cases were initially analyzed as one whole group. Logistic regression models were conducted to analyze associations between the initial and final radiographs in terms of categorical variables. Odds ratio and 95% confidence limits were calculated to compare the differences in categorical levels. The patients in the study who eventually underwent surgery after their last radiograph were further evaluated to deduce a probability estimate for years without surgery. Logistic regression, odds ratio, and probability estimate analyses were performed using SAS version 9.4 (SAS Institute, Cary, North Carolina), and the level of significance was set as p < 0.05.

The cases were then separated into three groups based on the time between initial and final radiographs: less than five years (Group 1), five to nine years (Group 2), and greater than nine years (Group 3). The three groups were analyzed for progression of HVA, IMA, MTP osteoarthritis, TMT osteoarthritis, sesamoid position, and round sign. One-way analysis of variance (ANOVA) testing was used to evaluate the starting population and initial-to-final radiograph progression in HVA, IMA, and sesamoid positions. Chi-square testing was used to evaluate the starting population and initial-to-final radiograph progression in MTP osteoarthritis, TMT osteoarthritis, and round sign. ANOVA and Chi-square analyses were performed using Python version 3.10 (Python Software Foundation, Delaware, United States).

A cumulative rate of progression (change in angle per time) of HVA and IMA was calculated by dividing the change in respective angle progression by the time elapsed between the initial and final radiographs. The cumulative rate of progression was calculated by considering all cases as one whole group once again. The rate of progression of HVA and IMA was calculated with a level of significance of p = 0.05 and associated 95% confidence interval.

## Results

All cases were first considered in one group when performing an odds ratio analysis. An increase of one sesamoid unit position over baseline was associated with a 0.85-degree increase in IMA. Moreover, a one-unit progression of sesamoid position at baseline raises the odds of MTP osteoarthritis progression at the follow-up visit by 2.14 (OR = 2.14, p-value = 0.0007, CI = [1.35, 3.83]). A HVA increase of one degree increased the odds of TMT osteoarthritis progression at the follow-up visit by a factor of 1.17 (OR = 1.17, p-value = 0.0005, CI = [1.07, 1.34]). Patients with MTP osteoarthritis at the initial visit have 3.77 times higher odds of round sign progression at their follow-up visit (OR = 3.77, p-value = 0.027, CI = [1.16, 13.13]).

The progression of HVA, IMA, MTP arthritis, TMT osteoarthritis, sesamoid position, and round sign for the three groups is outlined in Table 1. The three distinct groups were now considered based on the time between the initial and final radiographs. For HVA, the initial radiographs and the initial-to-final radiographic progressions were statistically the same (p = 0.55 and p = 0.92, respectively). For IMA, the initial radiographs and the initial-to-final radiographic progressions were also statistically the same (p = 0.74 and p = 0.35, respectively). Finally, for the sesamoid position, the initial radiographs and the initial-to-final radiographic progressions were statistically the same (p = 0.18 and p = 0.11, respectively).

**Table 1:** Initial and final radiograph characteristics. Osteoarthritis (OA) and round sign were qualitatively evaluated on whether they were present to begin with and whether they had progressed or appeared on the final radiograph. SD, Standard deviation; *, statistically significant; %, percent; HVA, hallux valgus angle; IMA, intermetatarsal angle; MTP, metatarsophalangeal; OA, osteoarthritis; TMT, tarsometatarsal.

	Group 1 (<5 years)		Group 2 (5-9 years)		Group 3 (>9 years)		
	n = 16		n = 28		n = 8		
*Initial Radiograph*							
	Mean	SD	Mean	SD	Mean	SD	p-value
HVA (degrees)	29.5	8.2	30.7	8.9	25.6	12.6	0.55
IMA (degrees)	12.7	3.5	14.4	3.9	13.7	4.1	0.74
Sesamoid position	4.3	1.5	5.2	1.4	4.8	1.2	0.18
	Number of Feet	% of Total	Number of Feet	% of Total	Number of Feet	% of Total	p-value
MTP OA	2	13	14	50	2	25	0.0046*
TMT OA	0	0	5	18	1	13	0.093
Round sign	4	25	8	29	3	38	0.82
*Final Radiograph*							
	Mean	SD	Mean	SD	Mean	SD	p-value
HVA (degrees)	33.1	11.3	34.5	10.4	30.2	13.0	0.92
IMA (degrees)	14.4	3.1	15.6	4.3	14.0	3.7	0.35
Sesamoid position	4.5	1.6	5.4	1.3	5.4	1.3	0.11
	Number of Feet	% of Total	Number of Feet	% of Total	Number of Feet	% of Total	p-value
MTP OA	2	13	10	36	4	50	0.034*
TMT OA	1	8	7	25	1	13	0.20
Round sign	4	25	10	36	4	50	0.42

TMT osteoarthritis, MTP osteoarthritis, and round sign between the three groups were compared next. For TMT osteoarthritis, the initial radiographs and the initial-to-final radiographic progressions were statistically the same (p = 0.093 and p = 0.20, respectively). Likewise, round sign for the initial radiographs and the initial-to-final radiographic progression showed no difference (p = 0.82 and p = 0.42, respectively). Starting MTP osteoarthritis was different between the three groups (p = 0.0046).

To determine the rate of HVA and IMA progression, all cases were once again considered as a single group. The linear rate of HVA progression was determined to be 0.66 degrees per year (p-value = 0.05, 95% CI = [0.40, 0.93]), and the linear rate of IMA progression was 0.21 degrees per year (p-value = 0.05, 95% CI = [0.09, 0.32]).

Of the 52 individuals in the study, 24 had surgery after the final radiograph was captured. By analyzing the timing of the initial radiograph and eventual surgery, a probability estimate for years without surgery is shown in Table 2 (p-value = 0.05). About 6.88 years after the initial radiograph, 75% of the patients remained without surgery, and 8.15 years after the initial radiograph, 50% of the patients remained without surgery. Finally, 10.49 years after the initial radiograph, 25% of the patients remained without surgery.

**Table 2:** Probability estimates for years without surgery after obtaining the initial radiograph

Percent (%)	Point estimate (years)	Lower 95% confidence limit	Upper 95% confidence limit
25	10.49	8.5	11.89
50	8.15	7.22	10.49
75	6.88	5.42	8.01

## Discussion

Hallux valgus deformity is a complicated condition with many probable causes. Both intrinsic characteristics specific to the patient’s body and extrinsic forces that have acted upon the patient’s foot have shown a significant impact [6,7]. Because of potential impaired gait, infection, discomfort, pain, and possible unsightly perception, efforts have ensued to treat the condition and enhance understanding [2,20-22].

Angles of characterization, sesamoid position, round sign, and arthritic processes are all important parameters for the characterization of hallux valgus [5,23]. Some studies have been able to conclude that hallux valgus deformity progresses and has characterized prevalence in many distinct regions of the world [24,25]. Nonetheless, despite the extensive studies on the topic, there is still much to understand regarding hallux valgus and its progression.

In 2017, Koo et al. published an article on the progression of hallux valgus in Hong Kong [12]. The authors designed a retrospective study with 43 cases from 38 patients to assess whether severe hallux valgus progresses over time. The study analyzed and compared the plain films of patients between presentation and surgery, concluding a significant difference in HVA (p = 0.040, t = -2.128) and no significant difference in IMA (p = 0.281, t = -1.095). Additionally, a significant correlation was reported between the time to surgery and HVA progression (p = 0.031), while no significant correlation was reported between the time to surgery and IMA progression (p = 0.195). This study is important to outline that hallux valgus does progress and characterizes exactly in the domain in which it does so. It is important to note that the study was restrictive in its generalizability to diverse populations as the authors studied a Chinese population in Hong Kong. Moreover, the study analyzes severe forms of hallux valgus that ended with surgery, which again does not carry over to the cases that are still considered severe but not enough to require surgical intervention.

More recently in 2021, Menz et al. published a population-based prospective cohort study with adults aged greater than 50 years in the United Kingdom [17]. Participants were invited to fill out a postal survey questionnaire at baseline and at seven years. Hallux valgus progression was identified in 719 (24.3%) participants' feet but was not associated with baseline factors. This study included many patients (an initial survey was sent out to over 5,000 individuals) in a diverse UK patient population. While the study is well designed, it relies on patients' input and does not include objective radiographic parameters for evaluation. The study explores the initial causes of hallux valgus, its incidence, and progression but not particularly any rates of progression and quantitative characterization of the condition.

Our retrospective study was designed to better quantify the progression of the deformity and arthritic features in hallux valgus. Similar to other studies, the results of our study suggest that hallux valgus progresses over time. Furthermore, our odds ratio analysis demonstrates the prognostic ability of sesamoid unit position, sesamoid position at baseline, valgus angle, and MTP arthritis in predicting IMA, MTP arthritis, TMT osteoarthritis, round sign, respectively.

There was no statistically significant difference among the three groups in sesamoid progression, suggesting that progression does not relate directly to the time elapsed. MTP osteoarthritis and TMT osteoarthritis progression showed no statistically significant difference among the three groups. Therefore, they do not relate directly to the time elapsed between the initial and final radiographs. Additionally, the Chi-square analysis for round sign yielded no statistically significant difference among the three groups: Round sign progression does not relate directly to the time elapsed between the initial and final radiographs. In addition, our study showed that HVA and IMA progressed in all three groups. Because the progression of HVA and IMA for the three groups was statistically the same, our study shows that progression does not relate directly to the time elapsed. This result prompted us to again pool our three populations into one cumulative population for calculating a cumulative rate of progression for HVA and IMA. Finally, an analysis of patients that ended up with surgical treatment allowed for a probability estimate for years without surgery after the initial radiograph. By analyzing the relationship between the initial radiograph date and surgery date, our study offers an estimate as to how long a patient’s hallux valgus condition can persist until surgical intervention is utilized.

Our study was limited by its retrospective nature. As a result, the data was restricted to chart review and patient radiographs. Radiograph analysis is intrinsically limited based on the software and measurement tools applied. Furthermore, radiographic measurements were done in a non-blinded manner with respect to which group patients would fall in and the time between the initial and final images. All outcomes are limited by the number of participants in this study. Additionally, our study analyzes small measurements that may be limited by measurement technique and patient positioning during radiograph capture. Future experiments should include a well-designed, multicenter, multigeographic, randomized control study, which utilizes more patients, more radiographs per time point, and more radiographic time points per patient.

## Conclusions

This retrospective study suggests that hallux valgus deformity progresses over time and depends on variables such as sesamoid position, HVA, and MTP osteoarthritis. While studies suggest that hallux valgus progresses over time, we offer added insight into how it progresses and the factors that play a role. Due to the limited number of patients and only two radiographs per patient, these results must be interpreted with caution. Nonetheless, surgeons and patients should consider the results when developing a treatment plan.
